# Molecular Biology of Retinal Ganglion Cells

**DOI:** 10.3390/cells9112483

**Published:** 2020-11-15

**Authors:** Béla Völgyi

**Affiliations:** 1 János Szentágothai Research Centre, University of Pécs, 7624 Pécs, Hungary; volgyi01@gamma.ttk.pte.hu; 2 Retinal Electrical Synapses Research Group, National Brain Research Program (NAP 2.0), Hungarian Academy of Sciences, 1051 Budapest, Hungary; 3 Department of Experimental Zoology and Neurobiology, University of Pécs, 7624 Pécs, Hungary

The main goal of this thematic issue was to bring both original research papers and reviews together to provide an insight into the rather broad topic of molecular biology of retinal ganglion cells (RGCs). The objective for such a collection of articles is given by the fact that RGCs are the output neurons of the vertebrate retina and, in addition to integrating information and passing it to target neurons in retinorecipient brain centers, they also perform a significant computation to encode signals into action potential trains. Since this mechanism requires the coordinated expression, activation, modulation, deactivation, and disintegration of molecules that partake in RGC signaling, the molecular constituents of the RGC intracellular molecular milieu play a functionally relevant role in terms of visual signaling and vision. 

In a review article, May [1] summarizes factors that majorly determine the diffusion efficiency of nutrients between supplying blood vessels and inner retinal neurons including RGCs. It has been put forward that three basic types of nutrition supply mechanisms exist, namely the choroidal nutrition type, a retinal nutrition type, and a mixture of these two. In addition, species-dependent differences in supply mechanisms have also been pointed out. 

Besides normal functioning, changes in the expression levels of certain molecules may induce pathological alterations in the retina, or their down- and upregulation is simply the consequence of a chain of molecular changes that occur during the development of a disease. One of the most common vision deficits is myopia, which is a substantial public health problem worldwide. It has been known that defocused images alter eye growth and refraction. In this issue, Feng Pan [2] shows that defocused images change the signaling of certain RGCs in the mouse eye, which may be the first step in the induction of myopia development. In addition, work from the same laboratory demonstrates that besides changes in individual RGC signals, synchronous activation is altered as well, as a result of image distortion by defocusing [3]. Another very common progressive condition is glaucoma, which is one of the leading causes of irreversible blindness in the world and remains a major public health problem. A review article in this thematic issue by Parsadaniantz and colleagues [4] collects converging experimental and clinical evidence showing that glaucomatous optic neuropathy shares common neuroinflammatory mechanisms with “classical” neurodegenerative pathologies. Therefore, new combinations of neuroprotective and immunomodulatory therapies may provide therapeutic potential to prevent blindness in glaucoma patients. 

Mechanical impacts or chemical changes may deteriorate the retinal tissue, even with no clear sign of any progressive retinal disease, thereby inducing disruptive changes in the retinal tissue and RGC function. One of the great challenges of modern neuroscience is to prevent such alterations via neuroprotective mechanisms and even to induce regenerative processes in the nervous tissue. Dulz and colleagues report that the combined administration of glial cell line-derived neurotrophic factor (GDNF) and ciliary neurotrophic factor (CNTF) conferred lifelong protection to injured RGCs [5]. Interestingly, the simultaneous administration of GDNF and CNTF stimulated an intraretinal axon growth that was significantly more pronounced than treatments with either of the factors alone. Norrin has also been known for its neuroprotective effects on RGCs following N-methyl-d-aspartate (NMDA)-mediated excitotoxic damage. The article in this issue by Kassumeh and colleagues [6] examines how leukemia inhibitory factor (Lif) is involved in this protective effect. The authors found that Müller cell gliosis, as well as endothelin 2 (Edn2) and fibroblast growth factor 2 (Fgf2) expression, was substantially blocked in Lif-deficient mice, indicating that Norrin exerts its protective effects through a Lif-dependent manner. Finally, Yang and colleagues studied the effects of optic nerve crush (ONC) injury on RGC death/survival [7]. The authors show that RGC susceptibility to ONC displays a cell type-specific variability and that all examined cell types showed a significantly higher susceptibility to NMDA excitotoxicity than following ONC.

Besides their functional or pathological relevance, some of the expressed molecules or their combination can also be utilized as neurochemical marker fingerprints to detect specific subtypes of the 20–30 morphologically identified RGCs. In this issue, the spatial expression pattern of Ca^++^- binding proteins (CaBPs) in the mouse RGC population was described by Kovács-Öller and colleagues [8]. The authors revealed a rather homogenous distribution of CaBP-expressing RGCs across the entire tissue and showed a considerable overlap in the expression of the proteins in the same cell populations. 

Taken together, the Special Issue “Molecular Biology of Retinal Ganglion Cells” collects a set of articles that further expand our view regarding the intracellular molecular mechanisms that may occur during normal functioning or induced when pathological changes set on in RGCs. Therefore, this present article collection will open new perspectives for both basic research and clinically relevant investigations in the future.
